# Circadian clock-coordinated hepatic lipid metabolism: only transcriptional regulation?

**DOI:** 10.18632/aging.100123

**Published:** 2010-02-17

**Authors:** Frédéric Gachon, Xavier Bonnefont

**Affiliations:** ^1^ Department of Pharmacology and Toxicology, University of Lausanne, Lausanne, CH-1005, Switzerland; ^2^ CNRS, UMR 5203, Institut de Génomique Fonctionnelle, 34094 Montpellier, France

**Keywords:** Circadian clock, Lipid metabolism, Unfolded protein response, IRE1α, autophagy, growth hormone

## Abstract

By regulating the metabolism of fatty acids, carbohydrates, and xenobiotic, the mammalian circadian clock plays a fundamental role on the liver physiology. At present, it is supposed that the circadian clock regulates metabolism mostly by regulating the expression of liver enzymes at the transcriptional level. However, recent evidences suggest that some signaling pathways synchronized by the circadian clock can also influence metabolism at a post-transcriptional level. In this context, we have recently shown that the circadian clock synchronizes the rhythmic activation of the IRE1α pathway in the endoplasmic reticulum. The absence of circadian clock perturbs this secondary clock, provokes deregulation of endoplasmic reticulum-localized enzymes, and leads to impaired lipid metabolism. We will describe here the additional pathways synchronized by the clock and discussed the influence of the circadian clock-controlled feeding rhythm on them.

Circadian clocks are operative in virtually all light-sensitive organisms, including cyanobacteria, fungi, plants, protozoans and metazoans. These timing devices allow their possessors to adapt their physiological needs to the time of day in an anticipatory way. In mammals, circadian pacemakers regulate many systemic processes, such as sleep-wake cycles, body temperature, heartbeat, and many physiological outputs conducted by peripheral organs, such as liver, kidney and the digestive tract [1]. On the basis of surgical ablation and transplantation experiments, it was established that the suprachiasmatic nucleus (SCN) in the hypothalamus coordinates most of these daily rhythms [2], probably through both synaptic connections and humoral signals [3]. Interestingly, self-sustained and cell-autonomous molecular oscillators do not only exist in pacemaker cells such as SCN neurons, but are also operative in most peripheral, non-neuronal cell types [4]. These peripheral oscillators participate in the circadian control

## Research Perspective

of animal physiology. During the past few years, analysis of animal transcriptomes with the DNA microarray technology showed that many aspects of physiology are directly controlled by the circadian clock through control of the expression of enzymes and regulators involved in these physiological processes [5,6]. Although the mechanisms involved in these regulations are not yet understood in detail, it is likely that transcription factors whose expression is controlled by the circadian clock are involved [7]. Based on these circadian transcriptome profiling studies it is commonly thought that circadian metabolism is mainly the consequence of circadian transcription and possible effects of circadian clock-controlled post-transcriptional regulatory mechanisms have been largely neglected.

Interestingly, most of the enzymes involved in liver metabolism are localized in the membrane of the endoplasmic reticulum (ER) of hepatocytes. The ER is a complex luminal network in which protein synthesis, maturation, folding, and transport take place. It has been previously shown that the ER of hepatocytes exhibits a circadian dilatation which is a sign of ER stress [8]. This ER stress triggers the unfolded protein response (UPR) which is a conserved adaptative response to cope with the accumulation of unfolded proteins in this organelle. When unfolded proteins accumulate in ER, three pathways are activated, IRE1α, PERK and ATF6, which lead to the nuclear translocation of the transcription factors XBP1, ATF4 and ATF6, respectively. These transcription factors activate in turn the expression of genes coding for proteins involved in peptide folding and degradation to limit the accumulation of unfolded proteins [9]. In this context, we have recently described the posttranslational regulation of liver enzymes through a circadian clock-coordinated 12-hours period rhythmic activation of the IRE1α pathway [10]. The observed rhythmic activation of the IRE1α pathway leads to the expression with a 12-hours period of the XBP1-regulated genes that are included in the 12-hours period genes described recently in mouse liver [11]. Persistent activation of the IRE1α pathway in circadian clock deficient *Cry1*/*Cry2* ko mice induced the downregulation of ER membrane localized enzymes, including HMGCR and SCD1, leading to a perturbed lipid metabolism in the liver of this mice. The decreased expression of these enzymes could be caused by activation of the ER Associated Degradation (ERAD), a process involved in the elimination of unfolded proteins inside the ER[12] regulated by the IRE1α-XBP1 pathway [13], which has been shown to induce the degradation of HMGCR and SCD1. In addition, IRE1α is a ribonuclease that can also induce endonucleolytic decay of many ER-localized mRNA including *Hmgcr* mRNA [14,15]. These two functions could contribute in parallel to the regulation of lipid metabolism by ER stress. Elsewhere, the IRE1α-XBP1 pathway controls also lipid metabo-lism through direct transcriptional regulation of the genes *Scd1*, *Dgat2* and *Acc2* involved in lipogenesis. As a consequence, liver-specific deletion of the *Xpb1* gene resulted in a dramatic reduction of plasma lipids [16]. Finally, it has been shown that ER stress induces the degradation of the apolipoprotein ApoB100 [17,18] and then blocks VLDL secretion [19], which might be responsible for the fat accumulation in the liver in tunicamycin-injected mice [20].

Interestingly, IRE1α activation has been recently linked to induction of autophagy through activation of the Jun-Kinase pathway [21]. In addition, a genomic screen in fly cells demonstrated that knocking down genes involved in protein folding inside the ER or in the UPR, including *Xbp1*, increases basal autophagy levels [22]. Autophagy is a survival pathway classically associated with adaptation to nutrient starvation [23] and, as UPR, autophagy presented a diurnal rhythm of activation in rodent liver [24,25]. This is of particular interest if we consider the fact that autophagy is linked to lipid metabolism through regulation of intracellular lipid stores [26]. As a consequence, mice with an adipose tissue-specific deletion of the *Atg7* gene, an important regulator of autophagy, present an important defect in lipid storage [27,28]. IRE1α-dependent rhythmic regulation of autophagy could then participates to the circadian clock-coordinated lipid metabolism in mammals.

The disturbed metabolism observed in *Cry1*/*Cry2* ko mice is probably responsible of the aberrant activation of the Sterol Responsive Element Binding Protein (SREBP) transcription factor, an ER membrane bond protein that, in low sterol conditions, translocates to the Golgi to be cleaved and released in order to migrate in the nucleus where it activates genes coding for enzymes involved in cholesterol and fatty acid metabolism [29]. It has been shown that the ER stress induced activation of SREBP1 and SREBP2 [30,31] correlates with the depletion of INSIG regulatory proteins probably through a decreased synthesis of the protein [32,33]. Interestingly, the circadian clock influences also the activation of the SREBP pathway through the control of *Insig2* mRNA expression [34]. Both transcriptional and post-transcriptional circadian clock-coordinated events seem to be involved in the rhythmic activation of the SREBP pathway.

**Figure 1.**
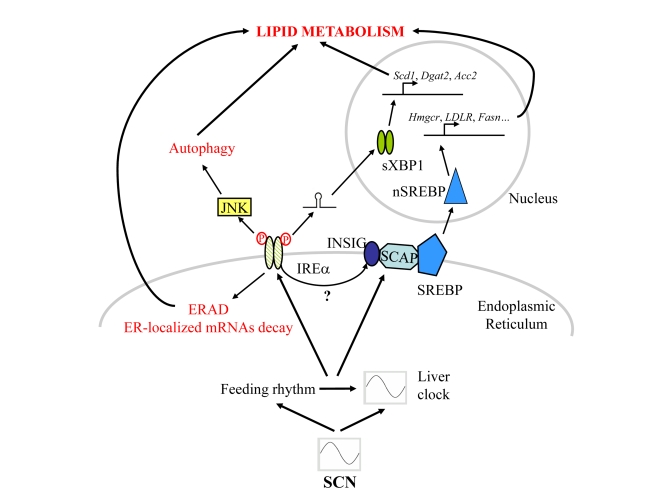
Schematic representation of the signalling pathways post-transcriptionally regulated by the circadian clock and/or rhythmic feeding cues in mouse liver.

As summarised in Figure 1, in addition to their rhythmic activation, all these pathways have in common the fact that they are regulated by feeding-fasting events. However, this feeding rhythm, like most behaviour, is also controlled by the circadian clock. To discriminate the genes dependant or not on a functional local circadian oscillator, this local clock has been inactivated in mouse liver. This strategy reveals that the expression of approximately 90 % of the rhythmic genes is dependent on a functional circadian clock and only 10 % is dependent on systemic cues [35]. However, the influence of feeding on rhythmic gene expression has been evaluated by a recent study which discriminates between gene induced by feeding and fasting. As expected, food-induced and food-repressed genes present a rhythmic expression which is shifted in response to a change in the feeding schedule [36]. More interestingly, this shift in the feeding schedule is able to induce rhythmic expression of food-regulated genes in the liver of *Cry1*/*Cry2* ko mice. These two studies raise the question of the differential influence of the molecular circadian oscillator and systemic cues on rhythmic gene expression: if these two signals can independently drive rhythmic gene expression, the circadian clock is able to fine-tune and modify feeding cues [34,36], whereas feeding cues can synchronize the molecular oscillator in peripheral organs [37].

However, feeding and food-regulated signals, as for example food regulated hormones like insulin, glucagon or leptin, did not represent the only circadian clock-regulated cues that can influence lipid metabolism. For example, the pituitary-secreted growth hormone (GH) has been shown to influence lipid metabolism in mouse liver. Long term excess GH secretion produces high serum triglyceride levels through stimulated lipolysis [38], whereas inhibition of GH signaling induced perturbed lipid metabolism resulting in liver steatosis [39], probably caused by reduced activation of HNF3β [40]. Moreover, the ultradian secretion patterns of GH are directly responsible for the sexually dimorphic expres-sion of several hepatic enzymes involved in steroids and fatty acids metabolism [41]. Interestingly, this dimorphism is impaired in *Cry1*/*Cry2* ko mice, with males exhibiting a feminized liver likely because of altered ultradian GH secretion in absence of a functional circadian clock [42].

During aging, the circadian system becomes much less responsive to entrainment by light [43,44], and displays loss of temporal precision and robustness [45-47]. Such alterations of the circadian clock likely drive attenuation of the diurnal rhythm in circulating leptin [48]. Pulsatile GH secretion is also dramatically impaired in elderly subjects [49-51], leading to modifications in GH-dependent liver metabolism that resemble those observed in clock-deficient animals [42,52]. Interesting-ly, the various UPR pathways also decline in the liver during aging [53], as well as autophagy [54]. In summary, many aspects of lipid metabolism that are regulated by the circadian clock exhibit profound changes when age increases, although the liver circadian oscillator appears preserved in aged rats [55]. These changes could thus at least partly originate from alterations of the network constituted of the central clock and other peripheral oscillators. In this respect, it is worth noting that mice bearing mutated alleles of the circadian genes *Clock* and *Bmal1* display signs of premature aging [56,57]. The complexity of systemic cues influencing rhythmic gene expression has thus been rising during the last decade and defining the influence of these different signals on rhythmic gene expression will be thus an exciting challenge for the following years.
